# Prognostic relevance of mixed histological subtypes in invasive breast carcinoma: a retrospective analysis

**DOI:** 10.1007/s00432-022-04443-x

**Published:** 2022-10-31

**Authors:** Anna Rechsteiner, Daniel Dietrich, Zsuzsanna Varga

**Affiliations:** 1 Department of Pathology and Molecular Pathology, University Hospital Zurich, Schmelzbergstrasse 12, CH-8091 Zurich, Switzerland; 2 Swiss Group for Clinical Cancer Research, Bern, Switzerland

**Keywords:** Breast cancer, Prognosis, Special histological subtypes

## Abstract

**Purpose:**

The prognostic and therapeutic power of special histological subtypes in breast cancer in pure form or in combination with other histological subtypes is still not established, and diagnostic guidelines are cautious regarding prognostic power based on the histological subtype alone. Therapy decisions are guided in most cases independently of the histological subtype and are directed by biomarkers and tumor stage. In this study, we analyzed a comprehensive large retrospective breast cancer cohort with a special focus on histological subtype (other than ductal non-special type or lobular carcinoma) and correlated pure or mixed histological forms with pathological tumor stage and overall disease-free survival.

**Materials and methods:**

A total of 827 breast cancer cases with pure or mixed special histological types were retrospectively analyzed. Survival information was available in 645 of 827 cases.

**Results:**

A total of 293 cases had pure forms, and 534 cases had mixed histological subtypes. The most common pure special types were mucinous (23.9%), micropapillary (21.2%), high-grade metaplastic (13%), male breast cancer (8.2%), cribriform (6.8%), metastases (6.1%), apocrine and papillary (each 5.46%), NST with medullary and clear cell pattern (up to 3.4%) and high-grade neuroendocrine carcinomas (2.7%). Mixed forms were most frequently encountered in NST carcinomas with micropapillary components (41.8%), followed by mucinous (9.93%) and cribriform (6.74%) mixed patterns. In univariate analysis, no pure form had prognostic relevance compared with any mixed form with the basic pure element. Pooling pure histological subtypes with tumor stage and age in a linear random-effects model, the cribriform subtype had the most favorable prognosis, while male breast cancer showed the poorest outcome (*p* < 0.001). All other frequent pure forms had intermediate prognostic power (*p* < 0.001).

**Conclusion:**

Our results show that the analyzed special histological breast cancer subtypes (other than ductal and lobular carcinomas) do not carry prognostic information alone, either in pure form or in any combination with other subtypes. Prognostic groups including special subtypes, however, can strongly stratify breast cancer if tumor stage, age and biomarkers are included in the prognostic measurements.

## Introduction

The special subtypes of breast cancer represent approximately 10% of breast cancers (Caldarella et al. 2013; Dieci et al. 2014; Han et al. 2020; WHO 2019). Considering that breast cancer is the most common cancer in women worldwide, the special subtypes are being diagnosed in a large number of total cases (WHO 2019). Lobular and no special type (NST) subtypes represent the majority of breast cancer histological subtypes and have well-established prognostic power and corresponding therapeutic implications. However, regarding special subtypes, the prognostic power of many subtypes, especially intermediate- and high-grade subtypes, is still controversial (Caldarella et al. 2013; Dieci et al. 2014; Han et al. 2020; WHO 2019). The WHO 2019 edition therefore recommends a different category to previously distinct entities (such as clear cell and medullary types of breast cancer) and places many special subtypes into the NST carcinoma diagnostic category with the corresponding special differentiation pattern (WHO 2019).

In this study, we addressed the question of how pure and mixed histological subtypes correspond to tumor and nodal stage and whether the histological subtype alone (in pure form or in combination) carries any prognostic relevance.

## Materials and methods

### Patient cohort

Breast cancer cases with the diagnostic category of special subtypes (other than pure NST and lobular subtypes) were retrieved from the diagnostic files of the Department of Pathology and Molecular Pathology of the University Hospital of Zurich. Cases between 2005 and 2015 were included in the study population. We identified 827 cases with pure or mixed special histological types. The average age of the cohort was 61.3 years. The mean tumor size was 26.3 mm. A total of 284 patients were nodal positive at the time of the first diagnosis. Patients were followed up on average for 67.9 months. Follow-up data were available in the University Hospital patient charts and the database of the Department of Pathology and Molecular Pathology of the University Hospital of Zurich. The time period followed up was from 2005 until 2020. In 645 of 827 cases, follow-up information was available. (182 were lost to follow-up). Clinical information including tumor stage, nodal stage and information on hormone receptor and Her2 status are summarized in Table 1.

**Table 1:** Patients’ cohort *n* = 827.Basic clinical information such as mean age, mean tumor and lymph node stage and available survival information

Parameter, total cohort, *n* = 827	Average	n/a
Mean age (years)	61.3	0
Mean tumor size (mm)	26.3	84
Nodal positive (in %)	41.64	145
Nodal positive (total number of cases)	284	145
Mean months till last seen (ex lost to follow-up)	67.9	182
Estrogen positive (in %)	81.7	4
Progesterone positive (in %)	77.7	4
Her2 positive (in %)	26	4

### Definition of histological subtypes, biological categories and pure forms

The original histological subtypes were concordant with the time current WHO classifications (editions 2008, 2012); however, for the study, we adjusted the nomenclature as recommended in the 2019 WHO classification (WHO 2019).

#### NST carcinomas with clear cell features, previously defined as clear cell carcinoma

Clear cell carcinoma cells contain either glycogen or lipids or resemble sebaceous glands of the skin adnexal structures, and the content of the clear cell phenotype can be visualized with special stains, such as PAS or Oil-red-O stains (WHO 2019). Regarding hormone status, progesterone receptors (PR) are mostly negative, and estrogen receptors (ER) are positive in 50% of cases (WHO 2019). According to the 2019 WHO recommendation, clear carcinomas behave prognostically similarly to NST carcinomas if matched by tumor size, grade and lymph node status (WHO 2019; Akagi and Jorns 2018; Varga and Caduff 1999; Varga et al. 2004) (Ma et al. 2014; Vranic et al. 2020). In this study, carcinomas with clear cell morphology such as sebaceous, lipid-rich or glycogen-rich (as diagnosed prior to the 2019 WHO classification) were included and pooled to NST carcinomas with clear cell features.

#### High-grade metaplastic carcinoma

Metaplastic carcinoma represents a heterogeneous group of high-grade carcinomas, encompassing spindle, squamous and mesenchymal differentiated elements. They are usually large tumors and are mostly triple negative. Lymph node metastases are less common; distant metastases, however, can occur. High grade metaplastic carcinoma has a poorer prognosis than other triple-negative NST breast cancers and responds more poorly to neoadjuvant chemotherapy (WHO 2019; Corso et al. 2021; McMullen et al. 2019; Morgan et al. 2020; Tadros et al. 2021). In this study, only high-grade variants were included, variants with more favorable prognosis (fibromatosis like carcinoma, low-grade adenosquamous carcinoma and some subsets of matrix producing carcinomas) were not included (WHO 2019; Ruddy and Winer 2013; Spreafico et al. 2020; Wang et al. 2021).

#### High-grade carcinoma with neuroendocrine features

High-grade neuroendocrine tumors are densely cellular and hyperchromatic and strongly express neuroendocrine markers such as chromogranin and synaptophysin. Most of them are ER- and PR-positive, and symptomatic hormone production is very uncommon. For diagnostic purposes, it is important to exclude neuroendocrine differentiated metastases to the breast, which are more frequent than breast cancer with neuroendocrine features (WHO 2019; Cloyd et al. 2014; Marchio et al. 2017; Righi et al. 2010; Wang et al. 2014) (Sapino et al. 2001).

#### NST carcinoma with medullary features

Medullary carcinomas, according to the 2019 WHO classification designated as NST carcinoma with medullary features, have round cells with high-grade nuclei and syncytial architecture. Lympho-plasmacytic stroma infiltration is characteristic. They are mostly triple-negative. Patients with BRCA1 mutation often have breast cancer with medullary features. The relatively favorable prognosis is mostly attributed to the abundant lympho-plasmacellular infiltrate and characteristic immunophenotype of CD8-positive cells rather than the morphology itself (WHO 2019; Cao et al. 2013; Huober et al. 2012; Pedersen et al. 1994; Rechsteiner et al. 2018).

#### Micropapillary carcinoma

Micropapillary carcinoma is built of morula-like clusters with reverse polarity divided by blank spaces. The majority of micropapillary carcinomas are PR- and ER-positive. In many cases, patients with micropapillary carcinoma present with lymph node metastasis at the time of the primary diagnosis (WHO 2019; Hao et al. 2019; Liu et al. 2015, 2021; Mahe et al. 2013; Tang et al. 2017).

#### Papillary carcinoma

Papillary carcinomas have a basic papillary morphology with fibrovascular cores. As they are very rare, there are few prognostic data about them. For diagnostic purposes, it should be considered that papillary differentiated metastases are more frequent than breast cancer with papillary morphology (WHO 2019; Hashmi et al. 2020, 2021; Pal et al. 2010).

#### Cribriform carcinoma

Cribriform-type carcinoma consists of arches of cells building cribriform spaces. This subtype is mostly ER and PR positive and is associated with a favorable resp. excellent prognosis (WHO 2019; Liu et al. 2021; Branca et al. 2017; Zhang et al. 2013).

#### Mucinous carcinoma

Mucinous carcinomas have uniform cell nests surrounded by extracellular mucin. It is usually ER and PR positive and HER2 negative. Pure mucinous carcinoma seems to have a good prognosis, whereas mixed mucinous carcinoma has a worse prognosis (WHO 2019; Bae et al. 2011; Saverio et al. 2008; Lei et al. 2016; Marrazzo et al. 2020).

#### Carcinoma with apocrine differentiation

Apocrine carcinoma is defined as tumors with large nuclei with PAS-positive cytoplasm or foamy cytoplasm and the expression of androgen receptors. This subtype is mostly ER- and PR-negative and is often Her2-positive. The prognosis is controversial depending on the cohort addressed; some studies report a similar, a better or a worse prognosis in comparison with stage- and age-matched NST carcinomas (WHO 2019; Saridakis et al. 2021).

#### Male breast cancer

Male breast cancer is rare and affects a slightly older age group. The histological subtypes are the same, with NST being the most common. The prognosis is similar to female breast cancer; however, male breast cancer is diagnosed more often in advanced tumor and nodal stage (WHO 2019; Ruddy and Winer 2013; Spreafico et al. 2020; Wang et al. 2021) (Yu et al. 2015). Although male breast carcinoma may represent other histological subtypes as NST carcinomas, the WHO 2019 classification defines this category as one separate biological entity (WHO 2019; Ruddy and Winer 2013; Spreafico et al. 2020; Wang et al. 2021).

#### Metastases

The most frequent metastases found in the breast are hematological malignancies, melanoma, carcinomas of the lung, ovary, prostate, kidney, stomach and carcinoid tumors. In about one-third, the diagnosis of a primary malignancy is made in the metastatic lesion to the breast. The prognosis is generally poor. (WHO 2019; Lee et al. 2020). Although metastatic lesions into the breast encompass several tumor entities from a variety of organs, the 2019 WHO classification designates this category as a separate biological entity (WHO 2019; Ruddy and Winer 2013; Spreafico et al. 2020; Wang et al. 2021).

#### Mixed forms

We identified 534 cases of mixed subtypes, which represents a variety of different patterns in combination with NST carcinomas the most, but scattered other combinations with other subtypes were identified as well. Altogether, we found 81 groups of mixed special subtypes, mostly with just 1–4 cases each due to the large range of possible combinations. The mixed types contained up to 5 different histopathological types. In the statistical analysis, we considered only mixed forms in combination with NST carcinoma as other scattered forms could not be pooled into individual subcategories.

### Statistics

In the statistical analysis, we included only patients (*n* = 645) with follow-up information.

SSPS software was used to estimate Kaplan–Meier curves and compare individual subtypes using log-rank tests.

SPSS Statistics was used for statistical analysis to compare the pure subtypes with all mixed subtypes containing this subtype. Groups were compared based on their overall survival times (months from first diagnosis until last documented appointment in the clinical charts or documented death) using Kaplan–Meier curves and log-rank tests. Apocrine, cribriform, papillary, metaplastic, micropapillary and mucinous subtypes were separately analyzed. To simplify the number of subtypes, a frailty model on subtypes was calculated. Frailties were classified into high- and low-risk groups. Kaplan–Meier curves of high- and low-risk patients are displayed.

Another goal was to classify the subtypes into risk categories with respect to the following four parameters pT, pN, pM and age. To achieve this, for each parameter, an adequate random-effects model was calculated to estimate the subtype effects on the parameter (linear mixed-effects models for pT, pN and age, generalized linear mixed-effects model for pM). The estimated random effects were dichotomized such as high = good risk and low = poor risk. With the four high/low variables, an overall score could be calculated indicating the number of good risks.

Survival was also defined as an outcome because of the low number of events, which was to be modeled and thus was not part of the scoring. Overall, a *p* value of < 0.05 was determined to be statistically significant.

The estimated random effects were dichotomized as follows: high = below median, low = above median (high is good risk, low is poor risk). With the four high/low variables, an overall score could be calculated.

## Results

### Frequency and clinical features of special types of breast cancer

The most common special subtypes observed were NST carcinoma with clear cell features, high metaplastic carcinoma, carcinoma with neuroendocrine features, NST carcinoma with medullary features, micropapillary carcinoma, papillary carcinoma, cribriform carcinoma, mucinous carcinoma, carcinoma with apocrine differentiation, male breast cancer and metastases. All samples with a mixed histopathological appearance containing a special type were included as well.

A total of 827 cases were analyzed, and there were 8–70 cases of each pure subtype. In total, there were 293 cases of pure forms. Other relevant pure types, such as myoepithelial carcinoma, adenoid cystic carcinoma, rare salivary gland type carcinomas and lymphoepithelial-like carcinoma, were excluded due to the small sample size (2–3 cases per category). There were up to 223 cases of each mixed subtype. Our analyses focused on the 11 most common pure subtypes in combination with other subtypes, because there altogether 81 groups of mixed special subtypes, mostly with just 1–4 cases each due to the large range of possible combinations, could not be stratified into a biologically reasonable category.

In some cases, the special subtype was first diagnosed in a recurrent lesion. Therefore, it cannot be excluded whether the special subtype existed in the primary tumor and was not diagnosed as such or occurred as a new component. As some initial diagnoses go back to the nineties, it is possible that the special subtype initially diagnosed was not included yet in the WHO and in other diagnostic guidelines.

All relevant histopathological parameters determined in the original report were collected for each case, such as ER, PR, Her2, and Ki67, according to the time current guidelines.

**Fig. 1:**
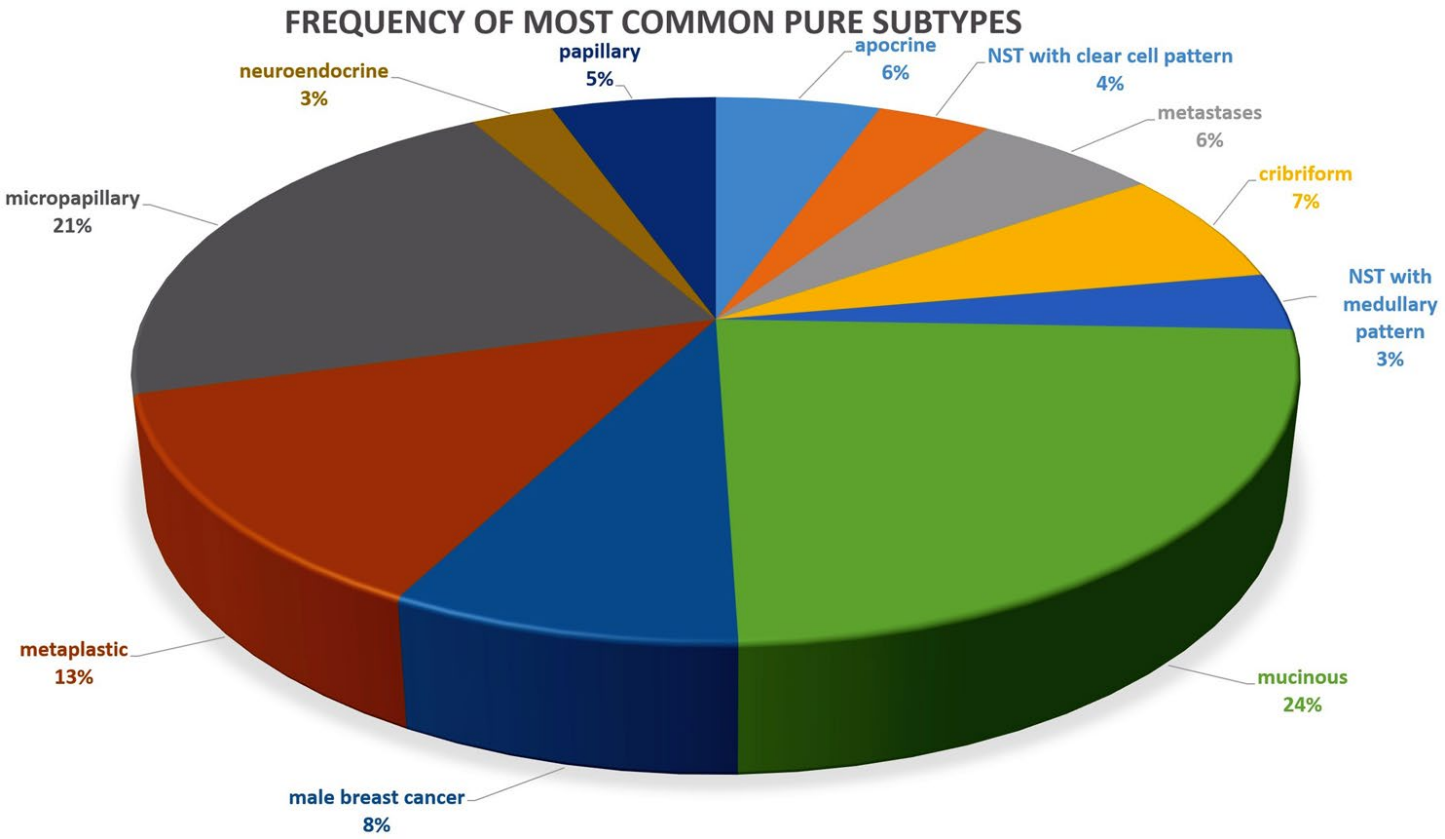
Graphical illustration of the most common pure histological subtypes

**Fig. 2:**
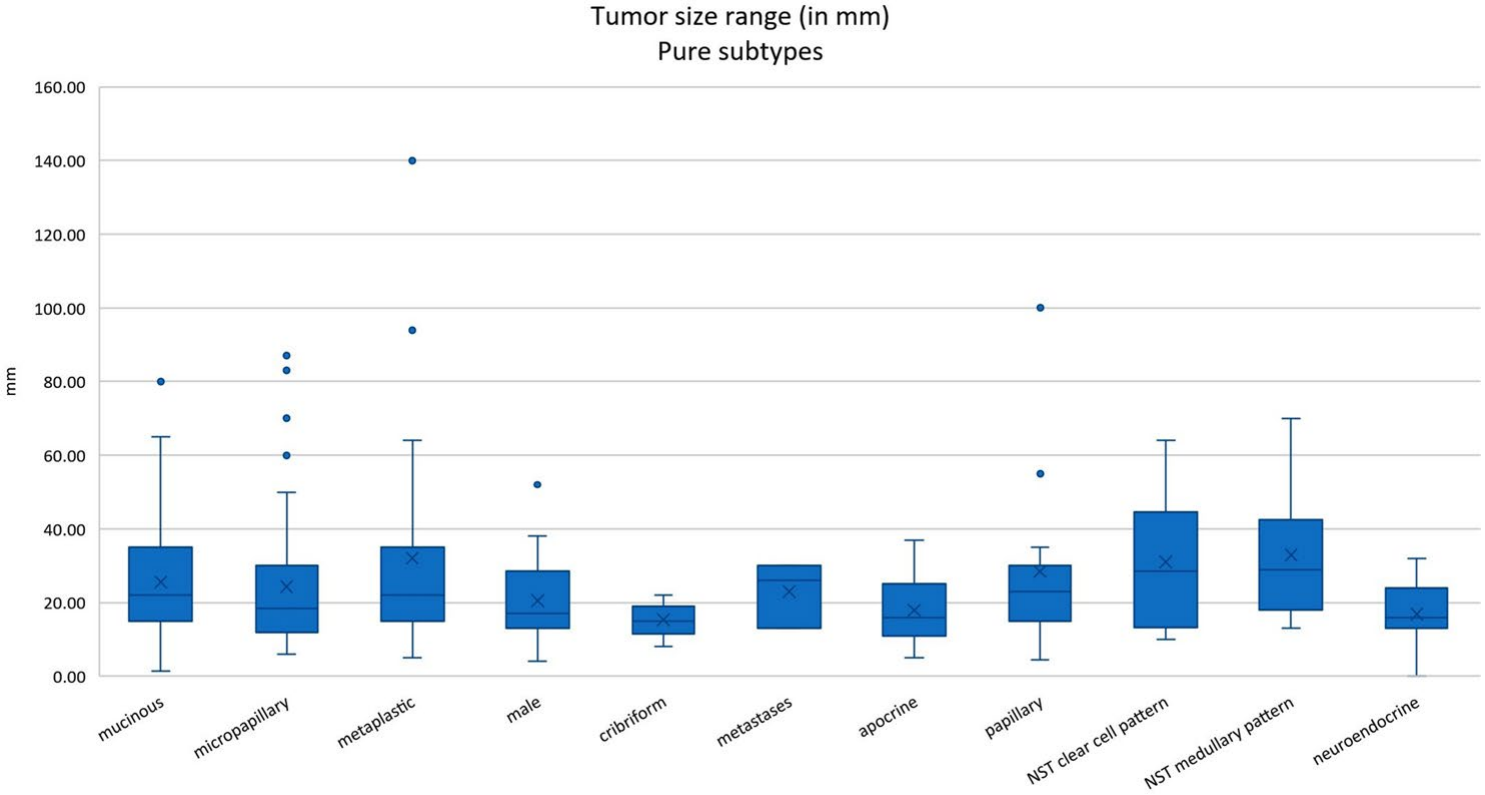
Tumor sizes (tumor status) in the most common pure histological subtypes

**Fig. 3:**
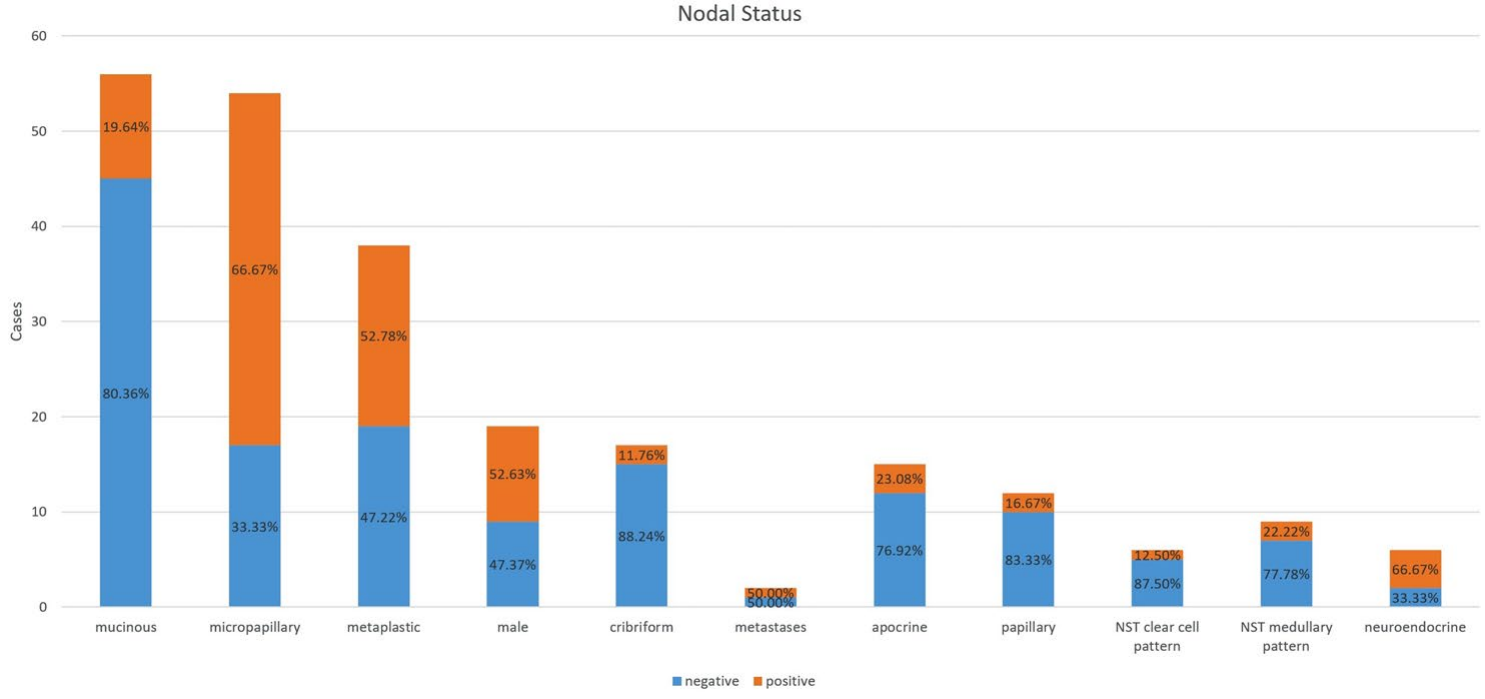
Nodal status in the most common pure histological subtypes

The frequency and clinical features of the most common pure histological subtypes are summarized in Tables 2 and 3 and are illustrated in Figs. 1, 2, and 3.

**Table 2:** Frequency of the most common pure histological subtypes

Subtype (*n* = 293)	Cases 2005–2015 (*n* =)	Percentage
Mucinous	70	23.89%
Micropapillary	62	21.16%
High-grade metaplastic	38	12.97%
Male breast cancer	24	8.19%
Cribriform	20	6.83%
Metastases	18	6.14%
Apocrine	16	5.46%
Papillary	16	5.46%
NST with clear cell features	11	3.75%
NST with medullary features	10	3.41%
High-grade neuroendocrine	8	2.73%

**Table 3:** Basic clinical information of pure histological subtypes such as mean age, mean tumor and lymph node stage and available survival information. na: not available

Subtype (*n* = 293)	Mean age (years)	Mean tumor size (mm)	Na	Mean involved axillary lymph nodes (*n* =)	Nodal positive (in percentage)	Na	Mean months overall survival	Lost of follow-up (*n* =)
Mucinous (*n* = 70)	64.5	25.6	7	11 of 56	19.64%	14	72.5	22
Micropapillary (*n* = 62)	61.4	24.3	4	34 of 52	65.38%	10	77.8	11
High-grade metaplastic (*n* = 38)	60.2	32	7	12 of 29	41.38%	9	50.8	8
Male breast cancer (*n* = 24)	65	20.5	3	10 of 19	52.63%	5	65.5	5
Cribriform (*n* = 20)	54.7	15.4	3	2 of 17	11.76%	3	60.5	5
Metastases (*n* = 18)	63.3	23	15	1 of 2	50.00%	2	39.7	3
Apocrine (*n* = 16)	64.4	17.9	1	3 of 13	23.08%	3	57.8	6
Papillary (*n* = 16)	70.7	28.4	1	2 of 12	16.67%	4	52.4	3
NST with clear cell features (*n* = 11)	64.4	31	3	1 of 8	12.50%	3	70.8	3
NST with medullary features (*n* = 10)	54.3	32.9	1	2 of 9	22.22%	1	53.2	1
High-grade neuroendocrine (*n* = 8)	59.1	19.7	2	4 of 6	66.67%	2	73.7	1

## Results

### Pure subtypes

#### Frequency

Regarding the pure subtypes, we identified 11 of the most common pure subtypes. The largest groups included mucinous carcinoma with 70 cases, micropapillary carcinoma with 62 cases and high-grade metaplastic carcinoma with 38 cases. The mucinous and micropapillary subtypes together account for almost half of the pure subtype cases. In total, there were 293 cases of pure subtypes in our cohort (Table 2).

#### Clinicopathological features

The mean age ranged from 54.3 years to 70.7 years, and the mean tumor size was between 15.4 mm (the lowest in the cribriform subtype) and 32.9 mm (the largest in the medullary and metaplastic subtypes). Axillary lymph nodes were involved between 11,76% (the lowest in the cribriform subtype) and 65–66% (the highest in micropapillary and high-grade neuroendocrine carcinomas). Overall survival was similar in all groups, ranging between 50.8 months (high-grade metaplastic carcinomas) and 77.8 months (micropapillary carcinomas).

**Fig. 4:**
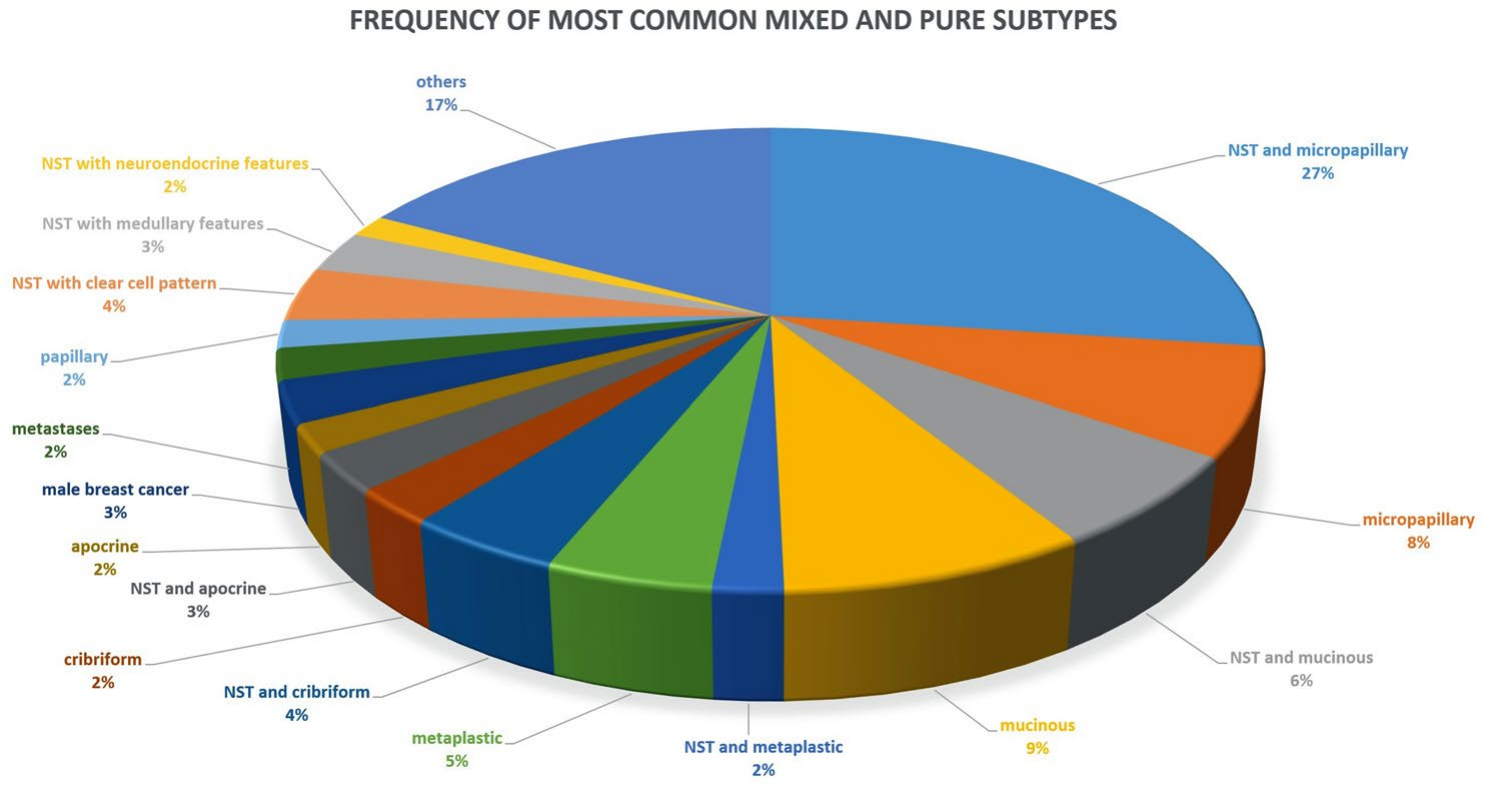
Graphical illustration of the most common mixed and pure histological subtypes

Details are shown in Table 3 and illustrated in Fig. 4.

### Mixed subtypes

#### Frequency

Within the mixed subtypes, there was a large variability of combinations and therefore a pronounced heterogeneity of cases. We identified 81 different groups with mostly only 1–4 cases each. Ductal with micropapillary and ductal with mucinous components were the highest numbers of cases. There were 223 cases of ductal subtype with micropapillary components representing the largest mixed special subtype group encompassing > 25% of the entire cohort. Ductal with mucinous and ductal with cribriform components were further frequent mixed subtypes, with 53 and 36 cases, respectively (6.4 vs 4.3%). The mucinous subtype was a common component in the pure and mixed subtypes.

Data with the most common mixed histological subtypes are shown in Table 4.

**Table 4:** Frequency of the most common mixed histological subtypes (as an accompanying component to NST)

Subtype (*n* = 417)	Cases 2005–2015 (*n* =)	in Percentage
Ductal, micropapillary	223	41.76%
Ductal, mucinous	53	9.93%
Ductal, cribriform	36	6.74%
Ductal, clear cell	25	4.68%
Ductal, apocrine	22	4.12%
Ductal, metaplastic	16	3.00%
Ductal, medullary	14	2.62%
Ductal, neuroendocrine	13	2.43%
Ductal, cribriform, tubular	8	1.50%
Ductal, papillary	7	1.31%

Clinicopathological features: as the number of mixed histological subtypes (in any combination) resulted in 81 groups, which succeeded the possibilities of any reasonable subgrouping, we included the summed up clinical-pathological features of the most common subtypes in pure form or in any other combination.

Data are shown in Table 5.

**Table 5:** Clinicopathological features of the most common histological subtypes, in pure form or in any combination with any other histological subtypes in the whole cohort

Subtype (*n* = 827)	Mean age (years)	Mean tumor size (mm)	n/a	Mean involved axillary lymph nodes (*n* =)	Nodal positive (in percentage)	n/a	Mean months overall survival	Lost to follow-up (*n* =)
Micropapillary (*n* = 325)	60.2	27	31	157 of 274	57.30%	51	69.7	64
Mucinous (*n* = 159)	64.4	29.9	41	48 of 120	40.00%	39	74.6	41
cribriform (*n* = 81)	60.2	19	6	21 of 69	30.43%	12	78.7	22
High-grade metaplastic (*n* = 61)	57.5	32.5	16	17 of 42	40.48%	19	53.8	15
Apocrine (*n* = 56)	61.8	25.5	5	27 of 50	39.02%	6	64	18
NST with clear cell features (*n* = 50)	62.2	31.5	5	16 of 41	39.02%	9	71.1	12
Papillary (*n* = 42)	66	29.1	3	13 of 34	38.24%	8	60.8	8
High grade neuroendocrine (*n* = 28)	66	25	3	9 of 22	40.91%	6	52.5	4
NST with medullary features (*n* = 25)	51.4	26.1	1	5 of 24	20.83%	1	56.7	2

### Prognostic relevance of pure and mixed subtypes

#### Univariate analysis of pure versus mixed subtypes

**Fig. 5:**
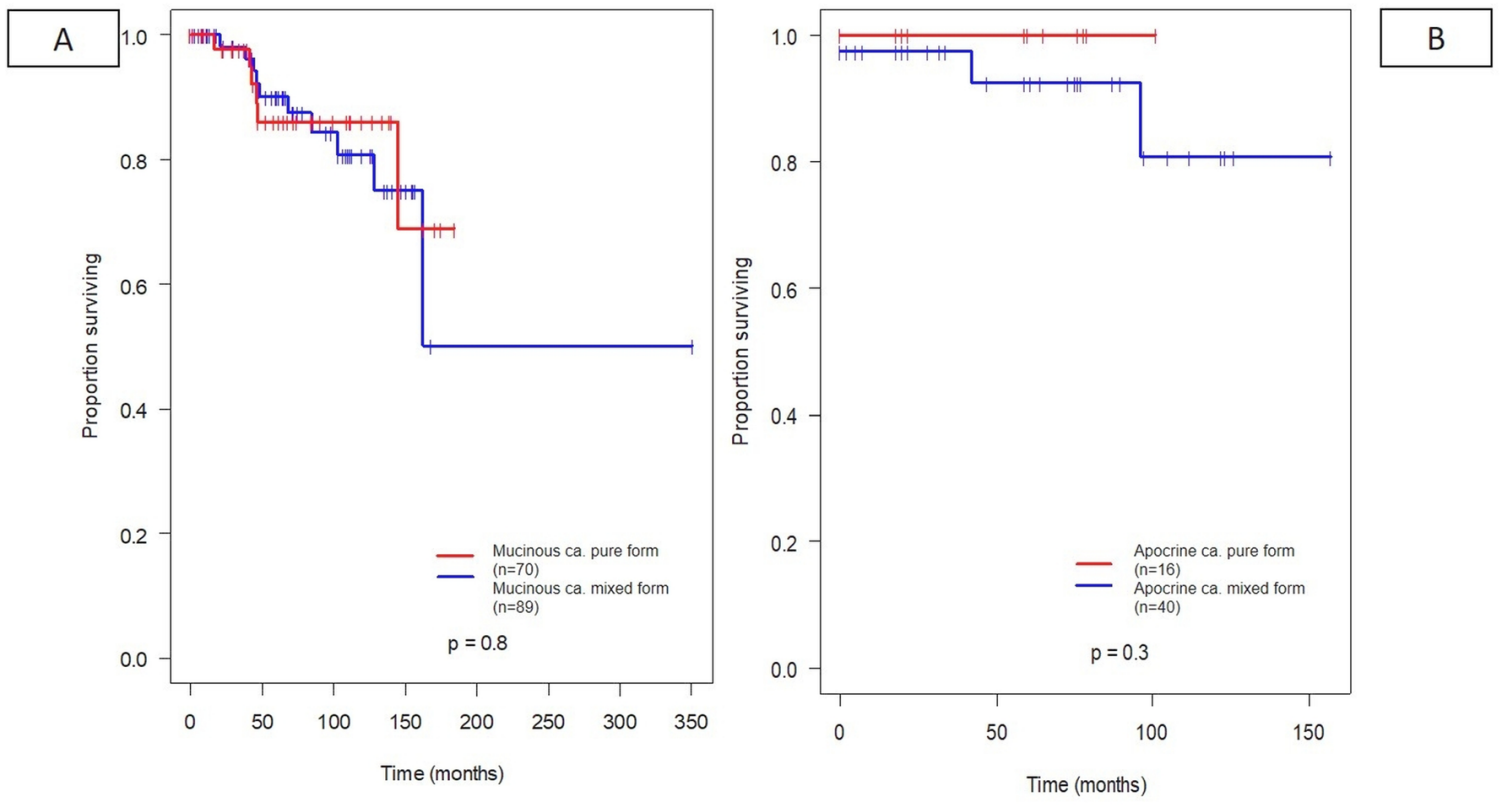
Prognostic value of the most common mixed histological subtypes (apocrine carcinoma (**A**), mucinous (**B**) carcinoma with or without additional NST or other components)

**Fig. 6:**
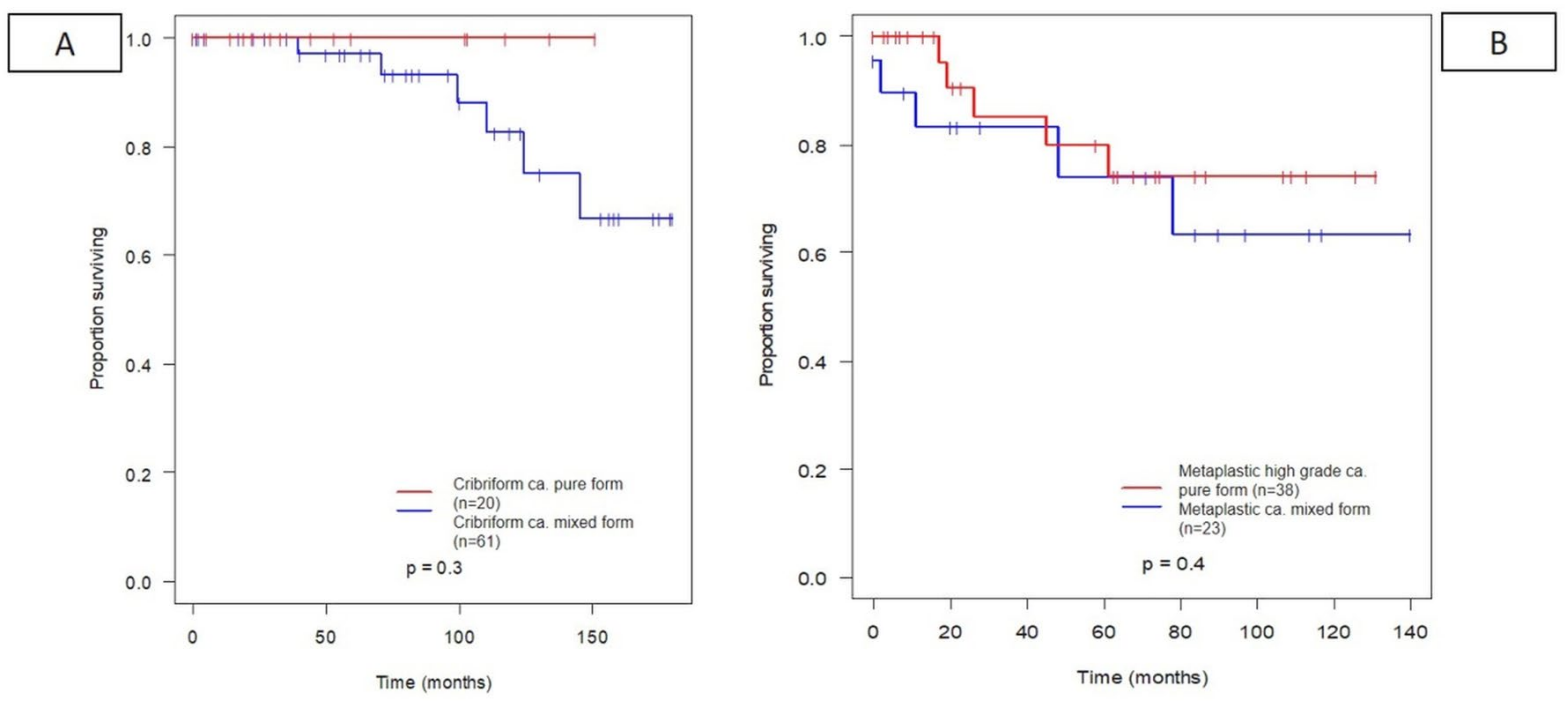
Prognostic value of the most common mixed histological subtypes (cribriform (**A**) carcinoma, high-grade metaplastic carcinoma (**B**) with or without additional NST or other components)

**Fig. 7:**
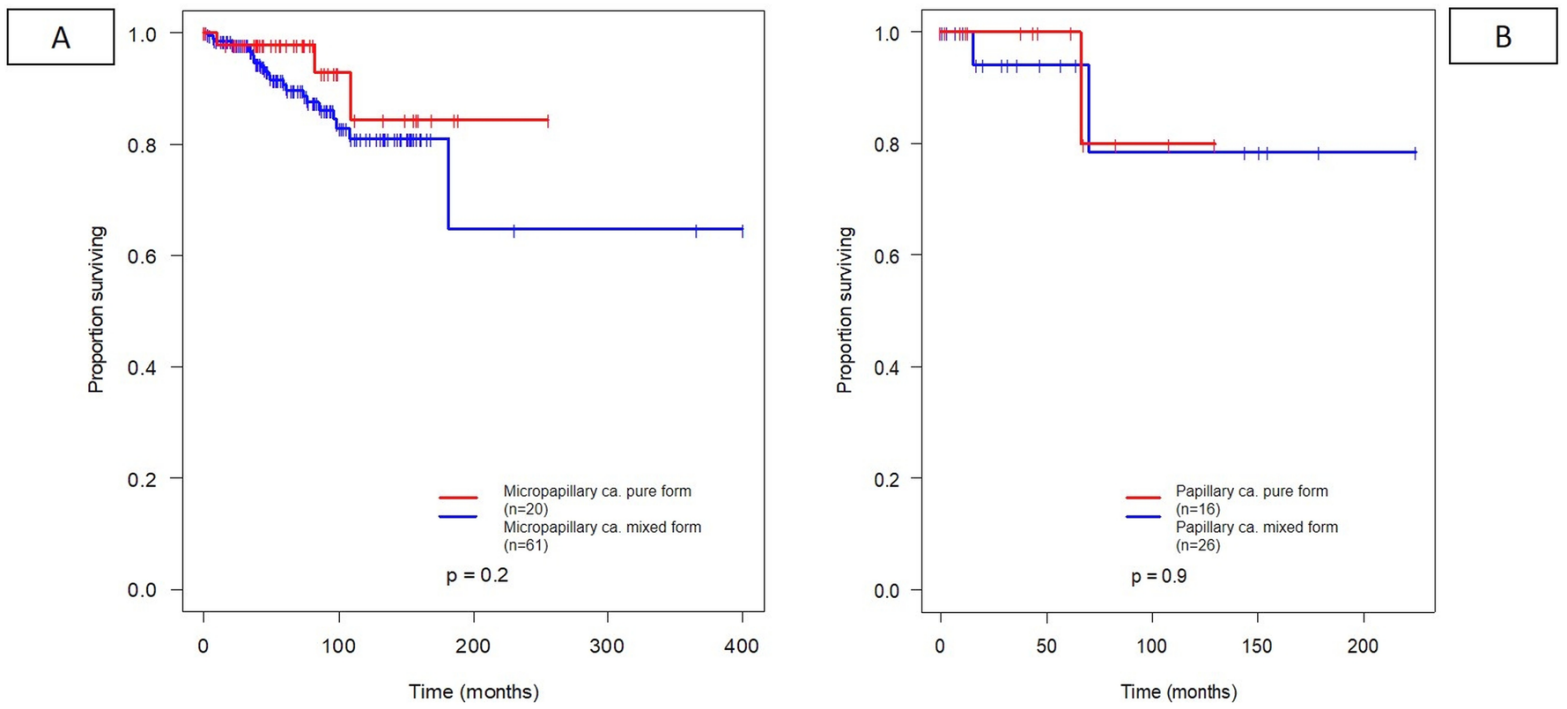
Prognostic value of the most common mixed histological subtypes (micropapillary (**A**) and papillary (**B**) carcinoma with or without additional NST or other components)

None of the most frequent pure subgroups in comparison with the mixed forms in any combinations (NST or other further histological subtype) had a significant difference in overall survival. Although there was a trend in all analyzed pure forms, such as mucinous, (Fig. 5A), apocrine (Fig. 5B), cribriform (Fig. 6A), high-grade metaplastic (Fig. 6B), micropapillary (Fig. 7A), and papillary (Fig. 7B) subtypes towards a more favorable overall survival, these differences did not reach statistical significance in univariate analysis.

#### Linear mixed random effect model by pooling tumor stage and age

**Fig. 8:**
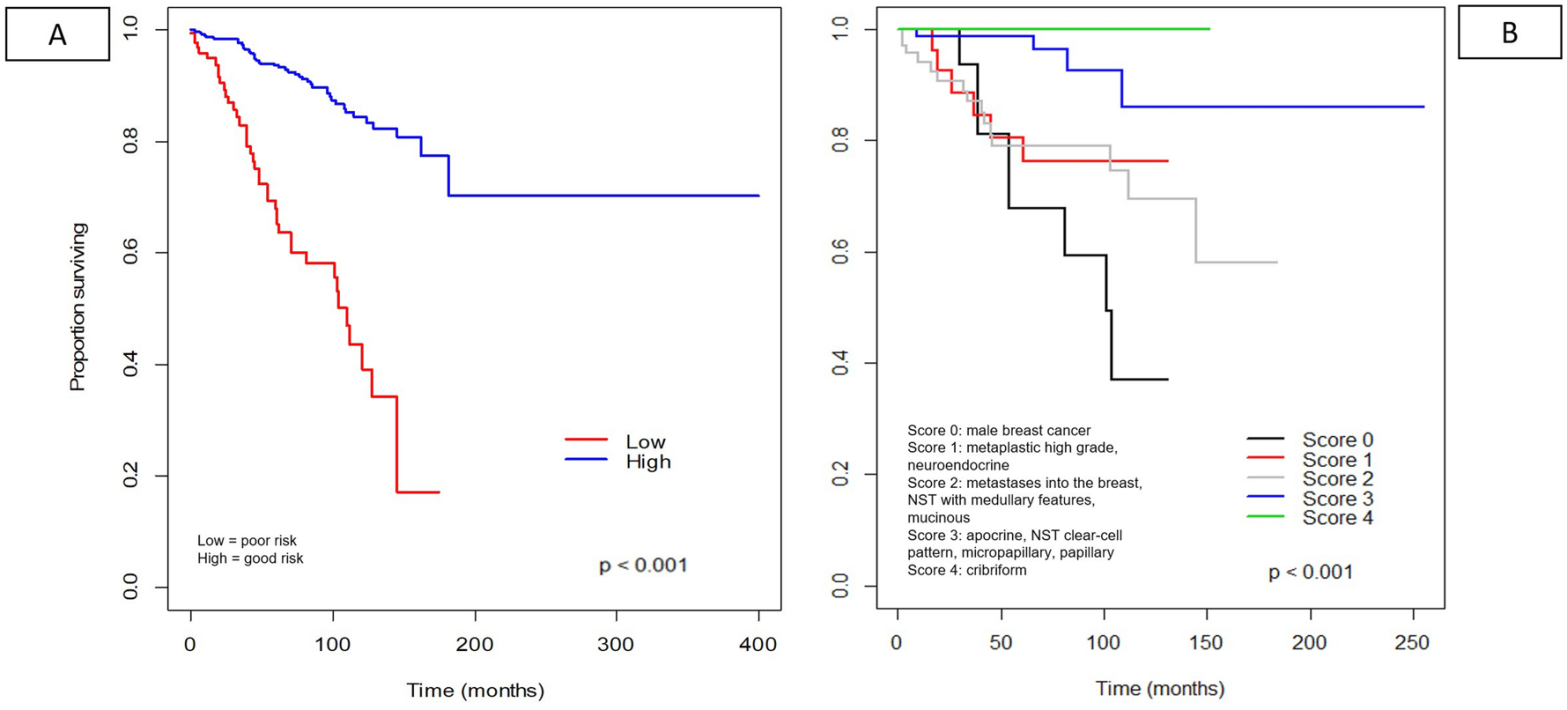
Random effects and prognostic groups (such as good or poor prognosis) included nodal stage (pN), tumor stage (pT), distant metastases (pM), and age. Figure 8A shows the results of a frailty model including all subtypes simplified in the low- and high-risk groups (**A**). Figure 8B shows the results of subtype scoring based on pT, pN, pM and age (**B**). Both results were highly significant (*p* < 0.0001)

Integrating tumor stage, nodal stage, distant metastasis status and age into all 645 cases with available survival data independent of the specific histological subtype, tumors could be divided into poor-risk and good-risk tumors. The results of the frailty model including all 81 subtypes simplified the risk into low and high risks, which were statistically highly significant, *p* < 0.001 (Fig. 8A). The results of the scoring considering histological subtypes could further stratify the pure forms into five prognostically distinct groups based on parameters such as pT, pN pM and age, which also had statistical significance, *p* < 0.001 (Fig. 8B). The most favorable prognostic group was associated with the cribriform pure subtype (score 4), and the poorest prognostic group was found with male breast cancer (score 0). Prognostic groups 1, 2, and 3 had intermediate prognostic power, including metaplastic high grade, neuroendocrine, metastases to the breast, NST with medullary and clear cell features, apocrine, micropapillary and papillary subtypes.

## Discussion

In this study, we showed that special histological subtypes in pure or mixed forms, especially in midrange and high-grade breast carcinomas, do not carry strong prognostic information based on the histological subtype alone. However, histological subtypes can be stratified into prognostically distinct subgroups if tumor and nodal stage as well as age are included in the survival curves. In our analyses, the cribriform subtype proved to be the most favorable histological subtype, whereas male breast cancer was associated with the worst prognostic outcome. Further midrange and high-grade histological subtypes/biological categories such as high-grade metaplastic, neuroendocrine, metastases to the breast, NST with medullary and clear cell features, apocrine, micropapillary and papillary subtypes, were associated with intermediate survival curves.

Our data corroborate very well with the cautious interpretation of special histological subtypes alone and further support prognostic information as recommended by the 2019 WHO classification of breast tumors (WHO 2019).

An interesting finding in our cohort is that mixed subtypes represent a rather heterogeneous group of cancer cases consisting of more than 80 separate subgroups depending on the combination, which was present in the histological samples. A similar observation of pronounced heterogeneity in mixed special histological subtypes was reported by Han et al. earlier (Han et al. 2020). In this large database of more than 200,000 breast cancer samples, including more than 12,000 special subtypes, the overall prognosis of special subtypes was comparable to those of invasive NST types (Han et al. 2020). Similar to our results, prognostic stratification after including further clinico-pathological parameters, such as biomarkers and tumor stage, could select prognostically favorable (apocrine, NST with medullary features, classical adenoid cystic) and poor (high-grade metaplastic) subgroups (Han et al. 2020). The heterogeneous over- or underrepresentation of distinct subtypes in our cohort explains why cribriform and male cancer were found to be associated with the most favorable resp. poorest prognosis. Another large study by Dieci et al. identified prognostically distinct subgroups only if other parameters, such as biomarkers, were included in the analyses (Dieci et al. 2014). In this study, special subtypes could be pooled together and designated as typically hormone receptor positive, typically Her2 positive or typically triple negative, resulting in favorable, intermediate and poor prognostic groups (Dieci et al. 2014). A similar observation was reported by Caldarella et al., who could pool favorable histological subtypes (such as mucinous, cribriform, and tubular types) based on their luminal A intrinsic subtype, which was also different from those tumors with unfavorable histological subtypes based on more advanced tumor stage than based only on histology alone (Caldarella et al. 2013).

It is, however, important to note, that the follow-up period of our study (2005–2020) is rather long and during this observation period, several therapy regiments have been changed having relevant impact on overall survival and on the overall prognostic relevance of the pure histological subtypes. Certain subtypes (apocrine, micropapillary, cribriform, etc.…) still pose a dilemma regarding prognostic value of the morphological differentiation and is most likely to be considered together in the context of the tumor/nodal stage, grade and the expression of intrinsic subtypes (WHO 2019; Varga et al. 2004; Hao et al. 2019; Liu et al. 2015, 2021; Mahe et al. 2013; Branca et al. 2017; Zhang et al. 2013, 2017; Saridakis et al. 2021; Yu et al. 2010; Vranic et al. 2013; Vingiani et al. 2013; Nassar 2004; Imamovic et al. 2018).

Strength of the study is the large case number and the consequent histological work-up and criteria of special subtypes according to the time current classifications as the cohort consists of cases from a single-center pathology. Limitation of the study is the retrospective data analysis and long follow-up period especially in view of the heterogeneous treatment modalities over the 15 years of follow-up possibly interacting with the prognostic relevance of the subtype alone in this single center cohort.

## Conclusions

Histological subtypes in breast cancer represent distinct prognostic subgroups; however, prognostic power can be reliably prognosticated only if further factors such as biomarkers and tumor/nodal stage and age are additionally added in the assessment of survival and prognostic characteristics. Therefore, the combination with other factors is probably more relevant for prognostic power than the histological subtype alone.

## Data Availability

The datasets generated during and/or analysed during the current study are available from the corresponding author on reasonable request.
